# Weather elements and the risk of tuberculosis incidence in China from 2005 to 2019: a county-level large observational study

**DOI:** 10.7189/jogh.16.04012

**Published:** 2026-01-12

**Authors:** Qiao Liu, Xiaoqiu Liu, Yuhong Li, Yaping Wang, Hongliang Zhang, Jue Liu, Yanlin Zhao

**Affiliations:** 1 Department of Epidemiology and Biostatistics, School of Public Health, Peking University, Beijing, China; 2 National Key laboratory of intelligent tracking and forecasting for infectious diseases, Chinese Center for Disease Control and Prevention, Beijing, China; 3 National Center for Tuberculosis Control and Prevention, Chinese Center for Disease Control and Prevention, Beijing, China; 4 Institute of Environmental Medicine, Peking University, Beijing, China; 5 Department of Environmental Science and Engineering, Fudan University, Shanghai, China; 6 Key Laboratory of Epidemiology of Major Diseases (Peking University), Ministry of Education, Haidian District, Beijing, China; 7 Institute for Global Health and Development, Peking University, Haidian District, Beijing, China

## Abstract

**Background:**

Tuberculosis (TB) remains a major public health challenge in China. Although meteorological factors are known to influence its transmission, their nonlinear and lagged impacts across regions and seasons remain unclear. We quantified these effects using the most detailed national data set available and explored how climate information can enhance TB prediction and control.

**Methods:**

We conducted a nationwide ecological time-series study by integrating weekly TB surveillance data (2005–19) with high-resolution meteorological and air pollution models. We assessed associations between TB incidence and meteorological factors using negative binomial regression and distributed lag nonlinear models to account for nonlinear and delayed effects.

**Results:**

From 2005 to 2019, TB cases in China decreased from 1.23 million to 0.75 million (estimated annual percent change <0 across all regions), with the burden remaining highest in western and southern China. Higher weekly mean temperature (incidence rate ratio (IRR) = 1.33) and precipitation (IRR = 1.03) increased TB risk, while greater temperature differences (IRR = 0.96) and relative humidity (IRR = 0.92) had protective effects. Temperature effects peaked in summer (IRR = 1.80; *P* < 0.05). Lagged analyses showed that extreme high temperatures and high wind speeds initially suppressed, but subsequently elevated TB risk, while higher precipitation and humidity showed delayed risk effects.

**Conclusions:**

By integrating fine-scale epidemiological and meteorological data, our study adds to our knowledge on TB epidemiology by more accurately characterising climate-disease interactions and enhancing the predictive capability of risk models. The findings provide empirical evidence to support the development of risk stratification tools and guide the implementation of proactive, phased intervention strategies aimed at mitigating the persistent TB burden in high-risk regions.

As highlighted by the World Health Organization (WHO), tuberculosis (TB) remains a critical global health challenge, with 10.8 million new cases and 1.25 million deaths reported in 2023 [1]. While post-pandemic trends show stabilisation, global targets for curbing TB incidence remain unmet [1]. Regional disparities further compound the crisis, with a significant burden concentrated in South-East Asia, Africa, and the Western Pacific [1]. China, contributing 6.8% of the global TB burden in 2023 and facing a high burden of drug-resistant TB, has made progress in this sense, but is still far from achieving the WHO End TB Strategy targets [1,2].

Existing literature provides evidence of the impact of meteorological factors on TB incidence – a relationship increasingly accentuated by climate change, which alters patterns of temperature, humidity, and precipitation [3]. This occurs through three primary, yet complex theoretical pathways: biological mechanisms (*e.g.* temperature affecting *M. tuberculosis* viability and host immunity [4–7]); behavioural factors (*e.g.* winter-time indoor crowding promoting airborne transmission, leading to delayed spring/summer peaks [5,6]); and systemic factors (*e.g.* extreme weather events disrupting health care access or diagnosis [3]). Prior regional studies confirm this complexity, noting that low temperatures, high temperatures, and moderate humidity levels can all show inconsistent or delayed associations with TB risk, often peaking months after exposure [7–10]. However, how the variables and mechanisms underlying these associations differ across regions remains unknown. More specifically, there is a lack of high-resolution, long-term, and spatially explicit studies that rigorously quantify these dynamic associations across a vast and climatically diverse area. Research specific to China, a country characterised by substantial geographical variation and a high TB burden, is especially limited.

Despite this growing recognition, a critical gap remains in the literature: there is a lack of high-resolution, long-term, and spatially explicit studies that rigorously quantify these dynamic associations across a vast and climatically diverse area. Research specific to China, with its substantial geographical variation and high TB burden, also remains fragmented.

We aimed to fill this gap by investigating the relationship between meteorological factors and TB incidence using the largest available county-level weekly TB dataset in China (2005–19). Our analysis integrates high-resolution meteorological and air pollution covariates with advanced DLNM modelling and explicitly considers seasonal and regional heterogeneity [11–15]. This study would therefore provide the first nationwide, high-resolution, and seasonally stratified assessment of the dynamic effects of meteorological factors on TB incidence in China. By integrating air pollution covariates and advanced DLNM modelling, it offers novel insights into how climatic and environmental factors interact to shape TB dynamics. These findings could provide evidence for improving the precision of TB risk prediction and for developing climate-informed early warning systems and region-specific prevention policies aimed at accelerating TB elimination efforts in China and globally.

## METHODS

### Study design, period, and data sources

We conducted a nationwide ecological time-series study integrating weekly TB surveillance data and high-resolution environmental models across China. We selected the period from 1 January 2005 to 31 December 2019, as it encompasses the most recent complete cycle of China’s national TB control efforts prior to major disruptions (*e.g.* COVID-19 pandemic) and aligns with the robust simulation periods of the weather research and forecasting (WRF) and community multiscale air quality (CMAQ) models used for environmental exposure assessment.

We retrieved weekly county-level TB case counts and incidence rates from the China Center for Disease Control and Prevention’s National Infectious Disease Network Direct Reporting System. As TB is a statutory class B infectious disease in China, prompt reporting is mandatory (within 6–24 hours of diagnosis), which ensures the completeness and temporal accuracy of the surveillance data for time-series analysis.

### Meteorological and air pollution data

We derived meteorological data from the WFC model, version 4.1.2, and air pollutant data from the CMAQ model, version 5.0.2, with an improved SAPRC-11 photochemical mechanism [16–18] (**Online Supplementary Document**). We validated both the WFC and CMAQ models against observational data from the National Climatic Data Center and the China National Environmental Monitoring Center, respectively. The validation results confirmed that the WRF simulation performance is consistent with previous studies despite the 36 km horizontal resolution, while the CMAQ model effectively simulates pollutant concentration variations according to established criteria [19]. High-resolution environmental data were subsequently integrated at the county level (**Online Supplementary Document**).

We included the following meteorological variables: mean temperature (°C), weekly average temperature, difference in temperature (°C), difference between the weekly maximum and minimum temperatures, relative humidity (%), weekly average relative humidity, precipitation (mm), weekly total precipitation, mean wind speed (m/s), weekly average wind speed. highest wind speed (m/s), and weekly maximum wind speed. Air pollutant variables included daily average concentrations of CO, O_3_, NOx, PM_10_, PM_2.5_, and SO_2_.

While direct time-varying adjustment for county-level socioeconomic status and health care access is challenging in national ecological studies, we accounted for long-term trends and time-invariant regional characteristics by including fixed effects in our models and conducting stratified analyses by four geographical and administrative regions (Eastern, Central, Western, and Northeastern China). We classified seasons according to general meteorological standards: spring (March to May), summer (June to August), autumn (September to November), and winter (December to February).

### Statistical analysis

First, we performed descriptive statistics for environmental factors (mean, standard deviation) and TB incidence (annual and weekly median incidence rate). Then, we calculated the estimated annual percent change (EAPC) to assess the long-term trend of the weekly TB incidence rates using the formula *EAPC* = (*e^β^* − 1) × 100%, where β represents the slope coefficient from a linear regression of ln(rate) against the calendar year.

To explore the association between environmental factors and the incidence of TB, we employed a negative binomial generalised additive model (NB-GAM) integrated with the DLNM framework to quantify the potentially nonlinear and lagged associations between environmental factors and weekly TB incidence, accounting for overdispersion in the case counts. The core model structure for the overall and regional analysis was:

*log*(*E*(*Y_cwt_*)) = α + *CB*(*X_c,t_*; *lag*) + *NB-GAM*(*Z_c,t_*) + *FE_c_* + *FE_t_*

Here, *Y_cwt_* represents the weekly TB incidence count per 100 000 population in county *c*, week *w*, and year *t*. *CB*(*X_c,t_*; *lag*) is the cross-basis function generated by the DLNM framework for the environmental factor *X* (*e.g.* meat temperature). *Z_c,t_* are the covariates, including the remaining meteorological variables and the six air pollutant variables (CO, O_3_, NO_2_, PM_10_, PM_2_._5_, SO_2_), all of which were standardised. *FE_c_* represents provincial and regional fixed effects, which together control for time-invariant confounders at the provincial level and structural differences between the four major geographical regions (Eastern, Central, Western, Northeastern China). *FE_t_* represents year and season fixed effects, controlling for overall long-term trends, national policy changes, and other unmeasured annual variations.

For the DLNM, we set the maximum lag period to 12 weeks, based on the typical incubation period of *M. tuberculosis* and consistency with prior short-term TB epidemiological studies. We used natural cubic splines for the exposure-response relationship (three degrees of freedom), and a fourth-degree polynomial for the lag-response relationship (four degrees of freedom). These specific degrees of freedom were optimised based on minimising the quasi-Akaike information criterion across a range of plausible values, ensuring flexibility while avoiding overfitting. We set the median value of each meteorological factor as the reference point for calculating the incidence rate ratios (IRRs).

### Model validation and multicollinearity check

We performed residual analysis and examined the goodness-of-fit using the Akaike Information Criterion for comparing alternative DLNM specifications (*e.g.* testing different degrees of freedom). We assessed the sensitivity of the results to the model specification by varying the maximum lag period (*e.g.* 8 and 16 weeks) and the degrees of freedom for the lag-response function (*e.g.* three and five degrees of freedom).

We explicitly assessed multicollinearity among the six meteorological variables. The variance inflation factor for all variables was consistently low (range: 1.163–3.116), well below the conventional threshold of 5, and the maximum Condition Index was 3.578, confirming that severe multicollinearity was not present and the parameter estimates are stable (Figure S1 in the **Online Supplementary Document**).

All statistical analyses were performed using R, version 4.1.2 (R Core Team, Vienna, Austria). Key packages utilised include ‘dlnm’ (for constructing the cross-basis functions) and ‘MASS’ (for negative binomial regression). Statistical significance was set at *P* < 0.05.

## RESULTS

### Weekly national burden and trends of TB, 2005–19

**Figure 1:**
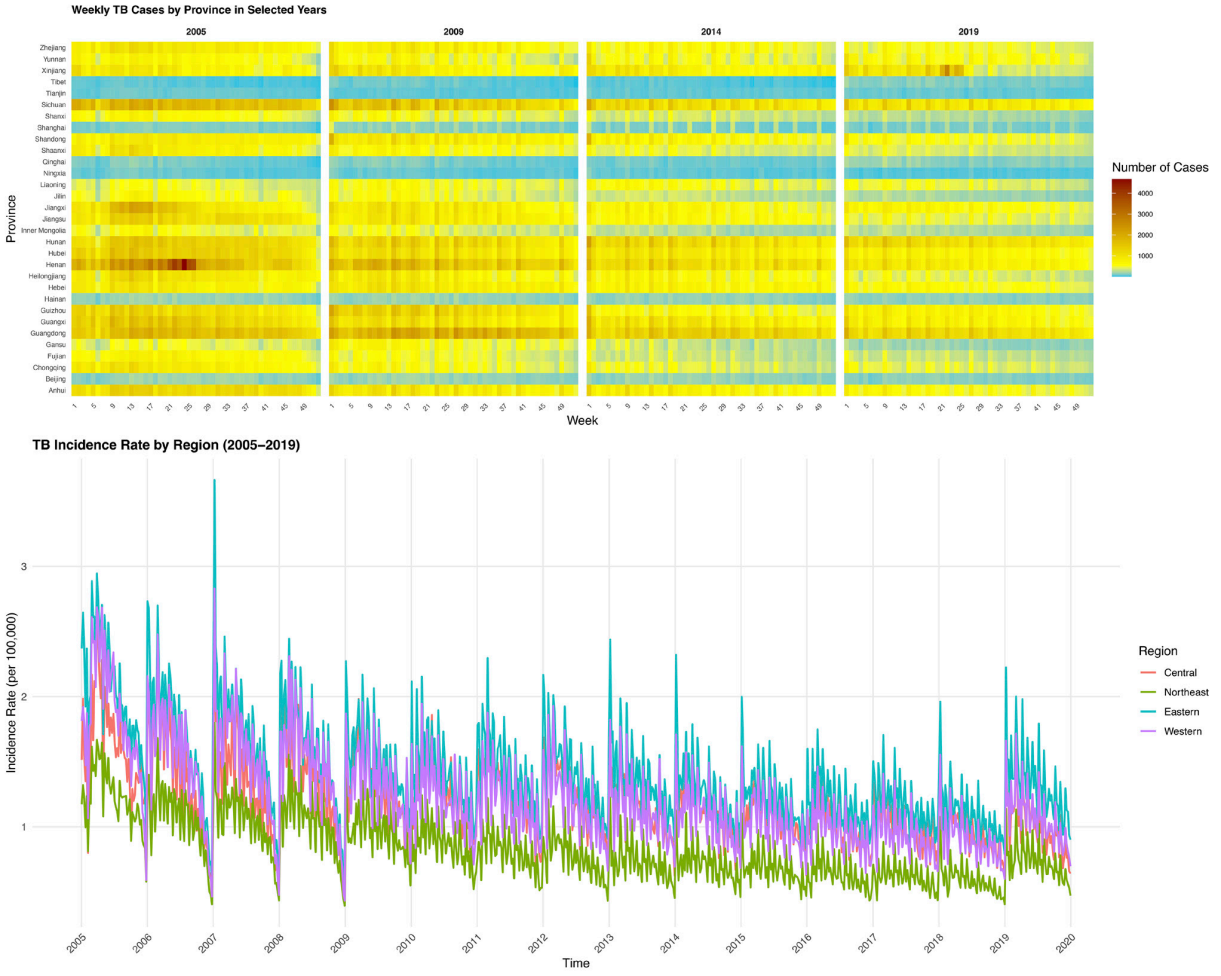
Weekly cases and incidence rates of tuberculosis in China, 2005–19.

The overall national burden of TB in China declined from 2005 to 2019 (**Figure 1**), whereby the total number of TB cases dropped substantially from 1.23 to 0.75 million, while the median weekly incidence rate decreased from 1.65 to 1.01 per 100 000 population. The timing of the annual incidence peak shifted over the study period, moving from week 13 in 2005 (peaking at 2.43 per 100 000 population) to week 1 in 2019 (peaking at 1.64 per 100 000 population).

### Regional and provincial heterogeneity

Although we observed a declining trend in TB incidence (EAPC<0) across the country, significant disparities in disease burden and the persistence of high incidence rates remained among China’s four geographical regions (Figure S2 in the **Online Supplementary Document**). Geographically, the Western region consistently reported the highest average annual TB case count (n = 330 402 cases), while the Northeastern region recorded the lowest (n = 78 842). We also noted geographical differences in the TB burden across China (Table S1 in the **Online Supplementary Document**). High-incidence provinces (Tibet, Xinjiang, and Hainan) maintained the highest median incidence rates, with Tibet consistently reporting around 4.4 per 100 000 population throughout the study period, while major municipalities like Shanghai and Tianjin reported the lowest rates. Regarding regional trends, the Western region consistently had the highest median weekly incidence rat, decreasing from 2.01 in 2005 to 1.32 per 100 000 population in 2019). The seasonal peak timing also varied across regions, with the Western and Central regions typically peaking early in the year (week 1), and the remainder between week 9 and week 14 (**Figure 1**).

### National distribution of weekly meteorological factors

We noted regional differences across China in terms of weekly meteorological factors. Northeastern China experienced the lowest winter temperatures (averaging −13.99°C), while the Eastern and Central regions recorded the highest summer temperatures (averaging 26.5°C). The weekly temperature difference remained relatively consistent across all regions, generally ranging from 13°C to 20°C. Weekly mean and highest wind speeds universally peaked in spring across all regions. The Eastern and Central regions received the highest precipitation in summer (up to 43.82 mm), aligning with peak relative humidity levels (38–44%) observed in summer across all regions (**Table 1**).

**Table 1:** Seasonal variations in weekly meteorological factors across Northeast, Eastern, Western, and Central China (2005–19), mean (standard deviation)

	Northeast	Eastern	Central	Western
	**Temperature in °C**			
**Spring**	5.77 (9.51)	15.78 (6.61)	15.68 (6.18)	12.45 (8.29)
**Summer**	21.89 (2.94)	26.58 (2.85)	26.44 (3.09)	21.91 (5.42)
**Autumn**	8.53 (8.5)	18.53 (6.43)	17.3 (6.15)	13.64 (7.65)
**Winter**	-13.99 (6.88)	4.93 (7.3)	3.56 (5.32)	0.95 (8.86)
	**Difference in temperature in °C**			
**Spring**	20.44 (4.56)	18.42 (6.15)	19.8 (4.05)	17.92 (4.13)
**Summer**	17.21 (5.58)	13.38 (6.36)	14.64 (5.04)	14.18 (5.37)
**Autumn**	18.51 (3.75)	14.7 (4.95)	16.49 (3.26)	14.47 (3.45)
**Winter**	19.17 (5.08)	15.47 (4.36)	16.62 (3.89)	15.82 (3.9)
	**Mean wind speed in m/s**			
**Spring**	4.95 (1.01)	4.12 (1.04)	3.67 (0.86)	4.07 (1.11)
**Summer**	3.57 (0.87)	3.41 (1.04)	3.01 (0.72)	3.2 (0.92)
**Autumn**	4.1 (0.91)	3.61 (1.31)	3.12 (0.71)	3.23 (0.93)
**Winter**	4.24 (1.01)	4.05 (1.38)	3.52 (0.81)	3.73 (1.14)
	**Highest wind speed in m/s**			
**Spring**	10.28 (2.26)	8.51 (2.32)	8.03 (2.11)	8.79 (2.5)
**Summer**	7.36 (1.92)	6.91 (2.21)	6.14 (1.59)	6.65 (2.13)
**Autumn**	8.46 (1.96)	7.4 (2.48)	6.6 (1.98)	6.83 (2.25)
**Winter**	8.44 (2.2)	8.12 (2.25)	7.43 (1.98)	7.68 (2.36)
	**Precipitation in mm**			
**Spring**	7.06 (11.84)	16.37 (31.39)	19.24 (33.27)	14.43 (24.05)
**Summer**	22.84 (27.78)	43.82 (68.93)	34.86 (51.67)	35.07 (49.04)
**Autumn**	6.86 (12.01)	14.38 (34.76)	10.53 (20.28)	14.69 (25.09)
**Winter**	1.56 (3.63)	5.44 (11.9)	5.4 (11.13)	3.53 (8.47)
	**Relative humidity**			
**Spring**	0.30 (0.09)	0.27 (0.09)	0.3 (0.07)	0.37 (0.14)
**Summer**	0.38 (0.08)	0.39 (0.12)	0.41 (0.12)	0.44 (0.13)
**Autumn**	0.27 (0.07)	0.32 (0.12)	0.28 (0.08)	0.32 (0.13)
**Winter**	0.21 (0.08)	0.24 (0.1)	0.21 (0.07)	0.25 (0.12)

### Overall effects of meteorological factors and air pollution covariates (non-lagged)

Negative binomial regression analysis, which controlled for fixed effects and air pollution covariates, identified several significant non-lagged associations between meteorological factors and TB incidence (all *P* < 0.001). Specifically, higher weekly mean temperature (IRR = 1.33) and precipitation (IRR = 1.03) were significantly associated with an increased TB incidence rate, acting as risk factors, while greater difference in temperature (IRR = 0.96) and higher relative humidity (IRR = 0.92) were found to be protective factors. This means that for every one standard deviation increase in mean temperature, TB incidence increased by approximately 33.0%, while a similar increase in relative humidity was associated with an 8.3% reduction in risk (**Table 2**).

**Table 2:** Associations between weekly meteorological factors and tuberculosis incidence rates by negative binomial regression

	IRR (95% CI)	*P*-value
**Weather elements**		
Mean temperature	1.330 (1.327–1.333)	<0.001
Difference in temperature	0.962 (0.961–0.964)	<0.001
Mean wind speed	1.002 (1.000–1.004)	0.076
Highest wind speed	1.008 (1.006–1.010)	<0.001
Precipitation	1.028 (1.027–1.029)	<0.001
Relative humidity	0.917 (0.916–0.919)	<0.001
**Air pollution**			
CO	1.067 (1.063–1.071)	<0.001	
O_3_	0.907 (0.905–0.908)	<0.001	
NOx	0.859 (0.856–0.861)	<0.001	
PM_10_	1.035 (1.029–1.041)	<0.001	
PM_2.5_	1.088 (1.082–1.095)	<0.001	
SO_2_	0.976 (0.974–0.978)	<0.001	

CI – confidence interval, IRR – incidence rate ratio

The analysis of air pollution covariates (**Table 2**), included in the model to adjust for potential confounding by air quality, also showed that these factors were significantly associated with TB incidence (*P* < 0.001). Elevated weekly average concentrations of CO (IRR = 1.07), PM_10_ (IRR = 1.04), and PM_2.5_ (IRR = 1.09) were associated with an increased TB risk, while an inverse association was noted for higher concentrations of O_3_ (IRR = 0.91), NO_x_ (IRR = 0.86), and SO_2_ (IRR = 0.98) (**Table 2**).

### Regional and seasonal eterogeneity of meteorological effects

**Figure 2:**
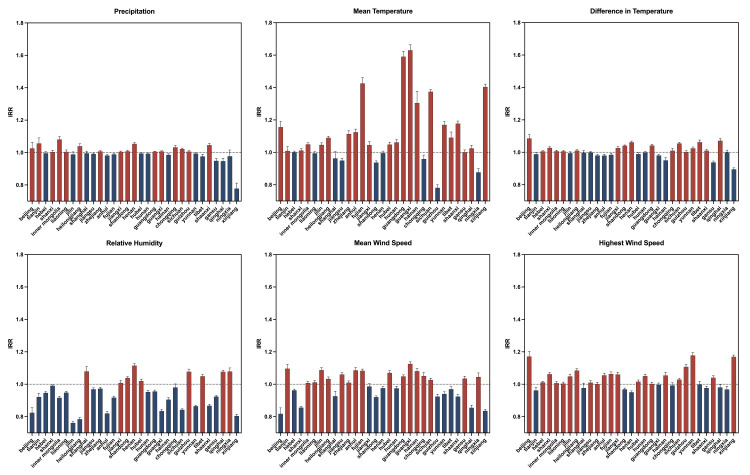
Spatial distribution of weekly meteorological effects on tuberculosis incidence in China.

The impact of meteorological factors varied substantially when stratified by region and season (Figure S3 in the **Online Supplementary Document**). Mean temperature consistently served as a significant risk factor across all seasons, but its positive effect was strongest in summer (IRR = 1.80), followed by autumn (IRR = 1.44) and winter (IRR = 1.36), with the smallest effect in spring (IRR = 1.20). The effect of mean wind speed demonstrated a seasonal shift, acting as a risk factor in autumn and winter (IRR = 1.05), but a protective factor in spring (IRR = 0.93). Precipitation showed its most significant risk effect in winter (IRR = 1.09). Provincial stratification indicated substantial heterogeneity: mean temperature had the strongest risk effects in southern coastal provinces like Guangxi (IRR = 1.63) and Guangdong (IRR = 1.59), while temperature difference was protective in the West (*e.g.* Xinjiang, IRR = 0.90), but a risk factor in the North (*e.g.* Beijing, IRR = 1.08) (**Figure 2**).

### Lag effect of meteorological factors on weekly TB incidence rates

**Figure 3:**
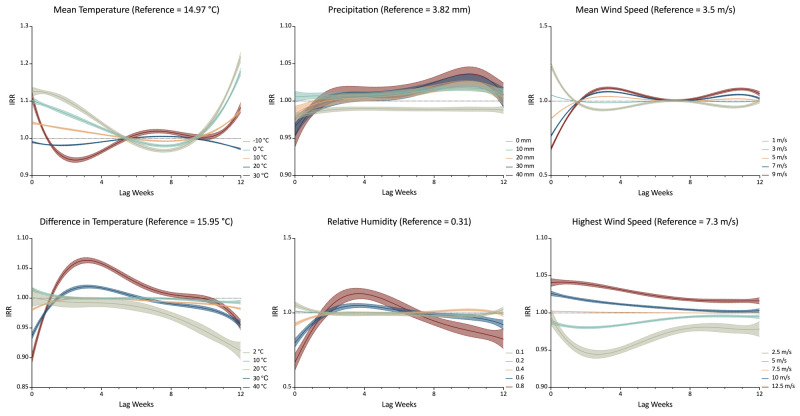
Lagged effects of meteorological factors on weekly tuberculosis incidence in China (2005–19).

The DLNM analysis showed nonlinear and lagged exposure-response relationships between meteorological factors and TB incidence. Compared to median exposure levels, extreme high temperatures (30°C) showed a brief initial protective effect (lag 1–5 weeks) before transitioning to increased risk, whereas extreme low temperatures (−10°C) demonstrated a delayed protective effect, emerging only after 5–10 weeks. For precipitation, higher rainfall (20 mm) initially acted as a protective factor but became a risk factor over extended lag periods. Elevated average wind speeds initially suppressed TB incidence, but became risk factors after a three-week lag, while higher maximum wind speeds consistently acted as risk factors throughout the lag period (**Figure 3**).

### Model validation and sensitivity analysis

The full models, which incorporated fixed effects and covariates, demonstrated strong explanatory power, with the residual deviance (ranging from approximately 2.43 million to 2.49 million) representing only 84.4–85.5% of the null deviance (2.92 million). This indicates that the models successfully captured a significant portion of the total variation in TB incidence. Sensitivity analyses further confirmed the robustness and stability of the primary findings.

## DISCUSSION

To the best of our knowledge, ours is the first assessment of the spatiotemporal heterogeneity and dynamic meteorological influences on TB incidence across China over a 15-year period (2005–19). Our findings confirm the significant progress in national TB control efforts, exemplified by a continuous decline in both case numbers and incidence rates. However, this success is unevenly distributed; we observed persistent and substantial geographic disparities and a distinct temporal shift in seasonality, with the peak incidence advancing from early spring (week 13 in 2005) to the beginning of the year (week 1 in 2019). Furthermore, common meteorological factors (mean temperature, temperature difference, precipitation, relative humidity, and wind speed) exert significant, immediate, and lagged effects on transmission dynamics, emphasising that climate is a critical, yet spatially heterogeneous determinant of China’s TB burden. The overall decline attributable to sustained national control programmes and socioeconomic progress is mathematically captured and controlled for by the inclusion of year fixed effects (*FE_t_*) in the model, allowing us to isolate and quantify the independent influence of meteorological variability on short-term TB incidence fluctuations.

Our finding that higher mean temperature is a significant risk factor aligns with established biological and behavioural pathways. Biologically, warmer temperatures are hypothesised to influence the host's immune response or affect the viability of *M. tuberculosis* outside the host [20]. Behaviourally, while warmer weather is associated with increased outdoor activities, the primary seasonal peak in early spring is widely hypothesised to be a delayed effect stemming from factors like winter indoor crowding, and subsequent vitamin D deficiency during the preceding cold months [12]. Conversely, the protective effect of relative humidity may be linked to the stabilisation of aerosolised droplets in moist air, causing them to settle quickly and reducing airborne transmission [21]. The complex shifts in wind speed effects – protective in spring, but a risk factor in autumn/winter – suggest that low wind speeds may allow for pathogen accumulation in crowded indoor environments, while high wind speeds (especially maximum wind speed) might facilitate long-range aerosol dispersion in outdoor settings [22,23]. The observed spatial heterogeneity in meteorological sensitivity confirms that a one-size-fits-all approach is insufficient; TB control strategies must be highly tailored to regional climate characteristics, population density, and health infrastructure [10,24,25].

The persistent regional disparities in TB incidence – particularly the stark contrast between the consistently high rates in Western provinces like Tibet and the low rates in coastal municipalities like Shanghai and Tianjin – strongly suggest the sustained influence of socioeconomic and health care resource inequalities. Prior studies have linked high TB burdens to inadequate housing, low income, and limited access to medical services, underscoring the role of social determinants in TB transmission and treatment completion [26,27]. Specifically, high-burden, underdeveloped regions face challenges related to health care accessibility and infrastructure, which are critical barriers recognised globally [28,29]. To achieve equitable TB elimination, policymakers must prioritise targeted investments in health care infrastructure and workforce distribution in high-burden, underserved regions to strengthen health system resilience and address the underlying social determinants of health.

The nonlinear and lagged effects quantified by the DLNM framework provide insights into the delayed nature of the infection cycle. The transient protective effect observed immediately following extreme high or low temperatures may reflect a short-term behavioural response (*e.g.* reduced human contact during extreme weather events) or delayed diagnosis, rather than an immediate change in biological risk [30]. The subsequent long-term increase in risk associated with high temperatures suggests a potential delayed biological impact on immunity or sustained changes in host susceptibility [31]. Similarly, the transition of precipitation from an early protective factor to a later risk factor indicates that immediate wash-out effects may suppress transmission, while persistent damp conditions at longer lags may promote pathogen survival in the environment. These temporal dynamics, which align with prior regional findings [20,32,33], underscore the need for public early warning systems that monitor meteorological forecasts up to 12 weeks in advance, targeting interventions based on the specific phase of the delayed risk.

### Strengths and limitations

Our study had several limitations. First, we could not utilise individualised data, but rather fine-scale county-level and weekly surveillance data sets only. Second, while we included environmental pollutants when adjusting for covariates, we lacked socioeconomic variables such as income and population characteristics, preventing us from exploring the full role of social determinants in TB incidence. Third, our results cannot be extrapolated to a global context. Nevertheless, our findings are valuable for understanding how environmental factors influence TB in a country with diverse climatic regions. Future studies could build upon this work by developing more comprehensive models that explore the relationships between meteorological factors, socioeconomic variables, and health policies.

## CONCLUSIONS

Our results indicate that, while the national TB burden in China is declining, regional disparities and complex, dynamic climate effects persist. Meteorological factors, meanwhile, exert significant, spatially heterogeneous, nonlinear, and lagged influence on TB incidence. These findings provide an advanced epidemiological framework for understanding climate-disease interactions, offering empirical evidence to support risk stratification tools and guide the implementation of proactive, phased intervention strategies aimed at mitigating the persistent TB burden in high-risk regions.

## Additional material


Online Supplementary Document
