# Supplementary material for: Weather elements and the risk of tuberculosis incidence in China from 2005 to 2019: a county-level large observational study
Source: J Glob Health. 2026 Jan 12;16:04012. doi: 10.7189/jogh.16.04012 (PMC12794371; doi:10.7189/jogh.16.04012)
Supplement: Online Supplementary Document [file jogh-16-04012-s001.pdf]

Supplement to: Liu Q, Liu X, Li Y, Wang Y, Zhang H, Liu J, Zhao Y. Weather elements and the risk of tuberculosis incidence in China from 2005 to 2019: a county-level large observational study. J Glob Health. 2026;16:04012.

Table of contents

Supplementary methods 2

Figure S1. Multicollinearity among the meteorological variables 6

Figure S2. EAPC of weekly tuberculosis incidence rate, 2005 to 2019. 7

Figure S3. Seasonal disparities in weekly meteorological effects on tuberculosis incidence in China 8

Table S1. Geographical differences in the TB burden across China. 9

Supplementary methods

1. Meteorological data sources

The meteorological data utilized in this study originates from simulations performed using the Weather Research and Forecasting model (WRF) version 4.1.2. The model applied the Final (FNL) Operational Global Analysis data from the National Centers for Environmental Prediction (NCEP) as meteorological input, with a spatial resolution of 1.0°×1.0° (https://rda.ucar.edu/datasets/ds083.3/, last accessed on March 2, 2023). The simulation period spans 21 years from 2000 to 2020, configured on a 197×127 grid with a horizontal resolution of 36 km × 36 km. Table S1 presents the detailed parameterization schemes of the WRF model.

Preprocessing of the meteorological data involved extracting WRF model output for each county-level administrative center in China based on their annual latitude and longitude coordinates from the original grid. Each county-level region corresponds to one grid cell's data. Subsequently, hourly data for each county were averaged to weekly data. For weeks with fewer than seven days of data, averages were computed based on the actual number of days available. Additionally, weekly maximum temperature, weekly minimum temperature, and weekly maximum wind speed were derived from the hourly data. The final weekly county-level dataset includes weekly maximum temperature, weekly minimum temperature, weekly average temperature, weekly maximum wind speed, weekly average wind speed, weekly precipitation, and weekly average relative humidity

2. Pollutant concentration data sources

To enhance the simulation performance, this study employed Community Multiscale Air Quality (CMAQ) version 5.0.2 with an improved SAPRC-11 photochemical mechanism[1-3], simulating the concentration changes of six pollutants ($PM_{2.5}$, PM_{10}, O_3, NOx (NO_2+NO), SO_2, and CO) in China from 2000 to 2020. The aforementioned WRF v4.1.2 was utilized to generate meteorology inputs. Anthropogenic emissions from 2008 to 2020 were sourced from the Multi-resolution Emission Inventory for China (MEIC) (http://www.meicmodel.org/, last accessed on July 2, 2023) [4]. Given the absence of emissions data in the previous MEIC v1.3 inventory before 2008, estimates for the years 2000–2007 were derived in this study based on emission ratios using the Emissions Database for Global Atmospheric Research data (EDGAR; https://edgar.jrc.ec.europa.eu/; last accessed in Jul. 2023) [5]. Anthropogenic emissions outside China were also derived from the EDGAR inventory. Both MEIC and EDGAR anthropogenic emissions encompass multiple sectors including power, industry, residential transportation and agriculture. Biogenic emissions were sourced from the Model of Emissions of Gases and Aerosols from Nature (MEGAN) v2.1 [6]. The processing of emission data in the model followed the approach by Wang et al. (2014) [7]. This method mapped NMVOC and PM2.5 emissions to the model species required by the SAPRC11 chemical mechanism and the AERO6 aerosol module. The Lambert projection was used to convert the original latitude and longitude data into emissions for each grid point (36 km) to fit the model input. Consistent with the WRF model, CMAQ also utilized a 197×127 grid configuration with a horizontal resolution of 36 km × 36 km, and the vertical grid was divided into 18 sigma levels, extending from the surface to ~20 km altitude. The first three days of model simulations were excluded to avoid the influence of initial conditions on subsequent predictions. Table S2 presents the detailed configuration of the CMAQ model.

Similarly, the preprocessing of pollutant concentration data was consistent with the method used for meteorological data. However, before obtaining the weekly county-level data, the hourly results from the CMAQ model were first averaged to daily values. The final dataset comprised weekly average concentrations of $PM_{2.5}$, PM_{10}, O_3, NOx, SO_2, and CO at the county level.

3. Observational Data and Model Validation

We validated the simulation results using observational data of meteorological conditions and pollutant concentrations. The meteorological observations including temperature at 2 m (T2), relative humidity (RH), wind speed (WS), and wind direction (WD) at 10 m were from the National Climatic Data Center (NCDC; https://www.ncdc.noaa.gov/, last accessed April 17, 2022).This dataset provided the meteorological observations at around 1,200 stations for the period from 2000 to 2020. The hourly concentrations of pollutants were validated using datasets from the China National Environmental Monitoring Center (CNEMC; http://www.cnemc.cn/, last accessed August 15, 2023). This dataset provided measurements from 1,600 sites across the country from 2014 to 2020.

Our previous article have detailed the validation results of the WRF-CMAQ model[8]. The results indicate that the WRF simulation results show slight deviations from the standard, primarily due to the relatively coarse 36 km horizontal resolution. Nonetheless, the model's performance is very close to that of previous WRF models and can provide accurate meteorological inputs for CMAQ[9-12]. The performance of the CMAQ model in simulating pollutant concentrations was evaluated using the criteria proposed by Emery et al. (2001) and EPA[13-15]. Comparison with observations shows that CMAQ effectively simulates the variations in pollutant concentrations. Overall, the model's simulation results are satisfactory.

Table S1. WRF scheme set-up

Microphysics	Thompson
Longwave Radiation	RRTM
Shortwave Radiation	Goddard
Surface Layer	Monin-Obukhov
Land Surface	Noah
Cumulus Parameterization	Grell-Devenyi
Planetary Boundary Layer	YSU

Table S2. CMAQ configurations and inventories input

Simulation time	2000-2020
Chemical transport model	CMAQ v5.0.2
meteorological model	WRF v4.1.2
resolution	36km × 36 km
chemical mechanism	SAPRC11
aerosol mechanism	AERO6
Meteorological inputs	FNL, 1.0° ×1.0°

Anthropogenic emissions	MEIC v1.3 (2008-2020)<br>EDGAR v5.1 (2000-2007)
Biogenic emissions	MEGAN 2.1

References

1. Ying, Q.; Li, J.; Kota, S. H., Significant contributions of isoprene to summertime secondary organic aerosol in eastern United States. *Environmental science & technology* **2015,** *49* (13), 7834-7842.

2. Hu, J.; Wu, L.; Zheng, B.; Zhang, Q.; He, K.; Chang, Q.; Li, X.; Yang, F.; Ying, Q.; Zhang, H., Source contributions and regional transport of primary particulate matter in China. *Environmental pollution* **2015,** *207*, 31-42.

3. Carter, W. P. L.; Heo, G., Development of revised SAPRC aromatics mechanisms. *Atmospheric environment* **2013,** *77*, 404-414.

4. Zheng, B.; Tong, D.; Li, M.; Liu, F.; Hong, C.; Geng, G.; Li, H.; Li, X.; Peng, L.; Qi, J., Trends in China's anthropogenic emissions since 2010 as the consequence of clean air actions. *Atmospheric Chemistry and Physics* **2018,** *18* (19), 14095-14111.

5. Crippa, M.; Solazzo, E.; Huang, G.; Guizzardi, D.; Koffi, E.; Muntean, M.; Schieberle, C.; Friedrich, R.; Janssens-Maenhout, G., High resolution temporal profiles in the Emissions Database for Global Atmospheric Research. *Scientific data* **2020,** *7* (1), 121.

6. Guenther, A. B.; Jiang, X.; Heald, C. L.; Sakulyanontvittaya, T.; Duhl, T. a.; Emmons, L. K.; Wang, X., The Model of Emissions of Gases and Aerosols from Nature version 2.1 (MEGAN2. 1): an extended and updated framework for modeling biogenic emissions. *Geoscientific Model Development* **2012,** *5* (6), 1471-1492.

7. Wang, D.; Hu, J.; Xu, Y.; Lv, D.; Xie, X.; Kleeman, M.; Xing, J.; Zhang, H.; Ying, Q., Source contributions to primary and secondary inorganic particulate matter during a severe wintertime PM2. 5 pollution episode in Xi'an, China. *Atmospheric environment* **2014,** *97*, 182-194.

8. Zhang, R.; Zhu, S.; Zhang, Z.; Zhang, H.; Tian, C.; Wang, S.; Wang, P.; Zhang, H., Long-term variations of air pollutants and public exposure in China during 2000–2020. *Science of The Total Environment* **2024,** *930*, 172606.

9. Shao, T.; Wang, P.; Yu, W.; Gao, Y.; Zhu, S.; Zhang, Y.; Hu, D.; Zhang, B.; Zhang, H., Drivers of alleviated PM2. 5 and O3 concentrations in China from 2013 to 2020. *Resources, Conservation and Recycling* **2023,** *197*, 107110.

10. Mao, J.; Li, L.; Li, J.; Sulaymon, I. D.; Xiong, K.; Wang, K.; Zhu, J.; Chen, G.; Ye, F.; Zhang, N., Evaluation of long-term modeling fine particulate matter and ozone in China during 2013–2019. *Frontiers in Environmental Science* **2022,** *10*, 872249.

11. Hu, J.; Chen, J.; Ying, Q.; Zhang, H., One-year simulation of ozone and particulate matter in China using WRF/CMAQ modeling system. *Atmospheric Chemistry and Physics* **2016,** *16* (16), 10333-10350.

12. Qiao, X.; Guo, H.; Tang, Y.; Wang, P.; Deng, W.; Zhao, X.; Hu, J.; Ying, Q.; Zhang, H., Local and regional contributions to fine particulate matter in the 18 cities of Sichuan Basin, southwestern China. *Atmospheric Chemistry and Physics* **2019,** *19* (9), 5791-5803.

13. Emery, C.; Tai, E.; Yarwood, G., Enhanced meteorological modeling and performance evaluation for two Texas ozone episodes. *Prepared for the Texas natural resource conservation commission, by ENVIRON International Corporation* **2001,** *161.*

14. Epa, U. S. *Guidance on the Use of Models and Other Analyses in Attainment Demonstrations for the 8-hour Ozone NAAQS*; EPA-454/R-05-002: 2005.
15. Epa, U., Guidance on the use of models and other analyses for demonstrating attainment of air quality goals for ozone, PM2. 5, and regional haze. *US Environmental Protection Agency, Office of Air Quality Planning and Standards* **2007**.

Figure S1. Multicollinearity among the meteorological variables

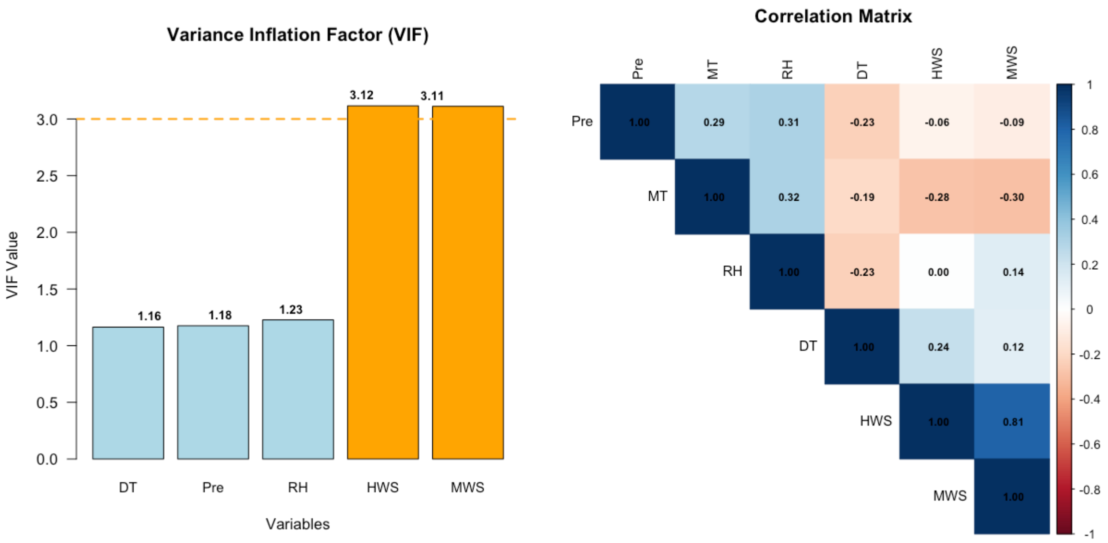

Figure S2. EAPC of weekly tuberculosis incidence rate, 2005 to 2019.

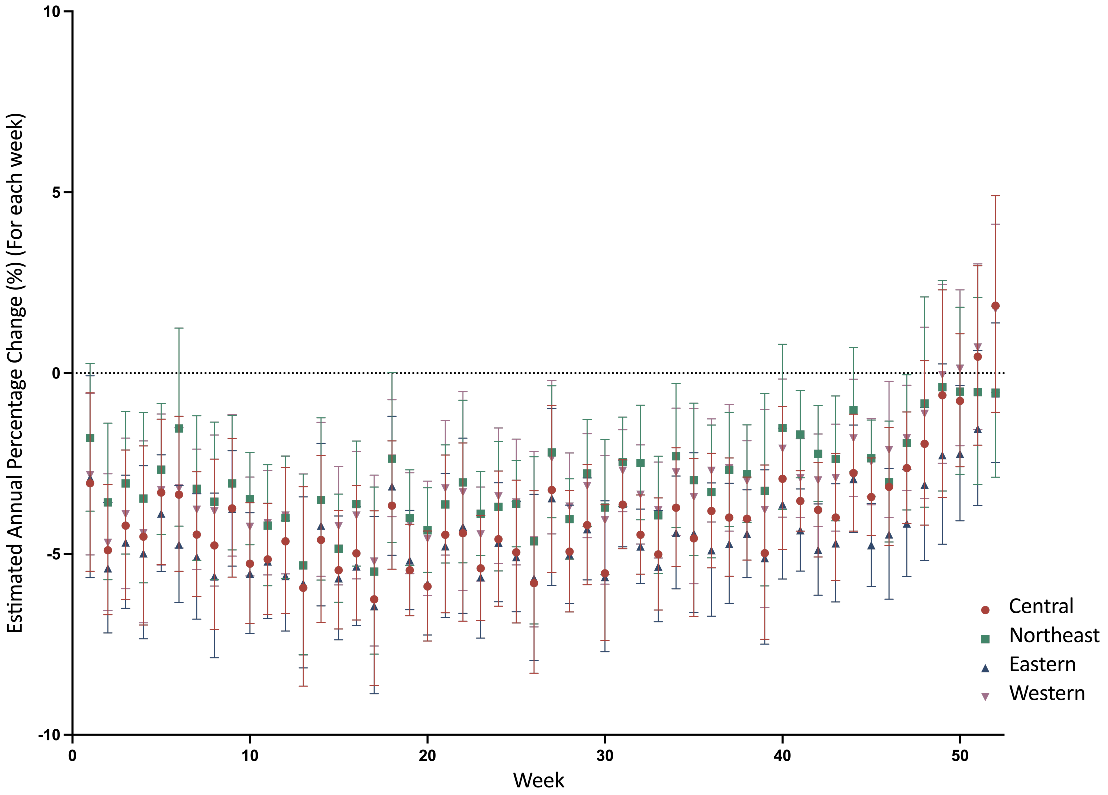

Figure S3. Seasonal disparities in weekly meteorological effects on tuberculosis incidence in China

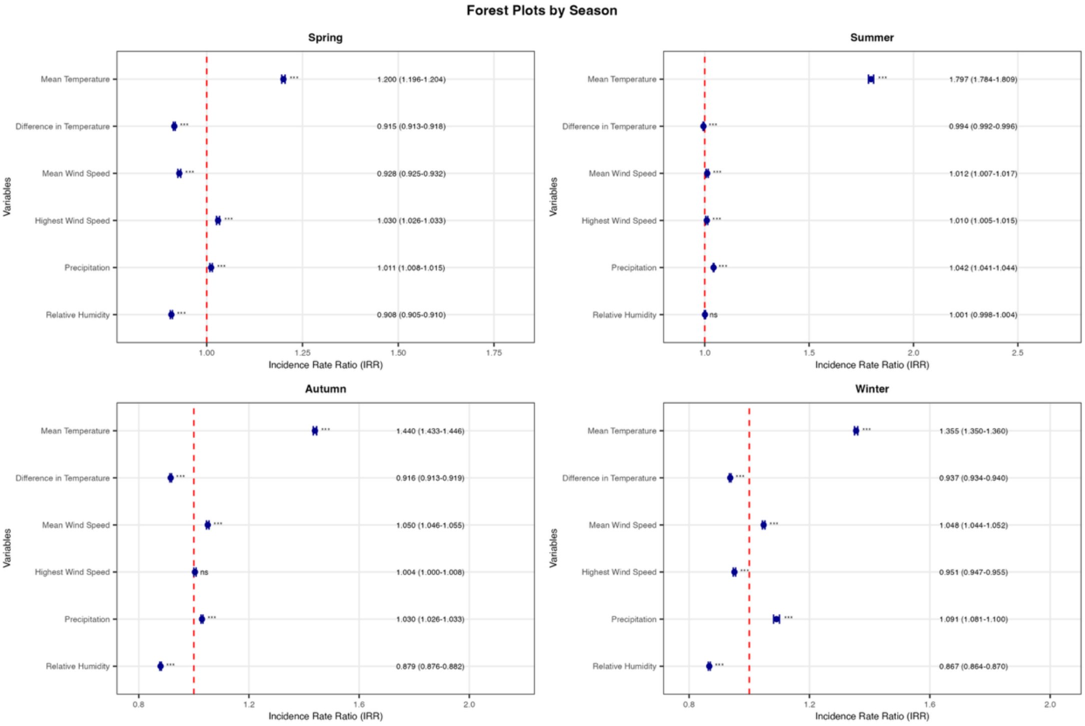

Table S1. Geographical differences in the TB burden across China.

Ranking	Provinces	Annual cases	Annual median incidence rates	Week of highest cases	Number of highest weekly cases	Ranking	Provinces	Annual cases	Annual median incidence rates	Week of highest cases	Number of highest weekly cases
Top cases						***Bottom cases***					
**Year 2005**											
1	henan	105404	1.7278	24	443	1	tibet	2750	4.3972	50	21
2	sichuan	88180	1.85865	47	110	2	ningxia	3650	1.2073	10	20
3	guangdong	82097	1.7899	31	204	3	tianjin	3922	0.7082	36	17
4	hubei	66089	2.1565	30	99	4	qinghai	5054	2.377	14	51
5	guangxi	65759	2.524	35	107	5	shanghai	6090	0.6138	9	30
**Year 2009**											
1	guangdong	95204	1.7723	9	101	1	tianjin	2965	0.4048	11	13
2	henan	80389	1.1175	10	96	2	ningxia	3442	1.0111	1	40
3	sichuan	72312	1.4294	23	140	3	tibet	3781	3.5575	1	21
4	guizhou	61144	2.10945	18	124	4	qinghai	5519	2.1078	19	22
5	hunan	59394	1.5343	49	73	5	shanghai	7492	0.403	1	86
**Year 2014**											
1	guangdong	67770	1.2457	14	74	1	tibet	2553	3.5854	16	14
2	henan	58582	0.8797	1	99	2	ningxia	3407	0.8444	37	31
3	hunan	57407	1.4082	1	107	3	tianjin	3447	0.32	22	15
4	sichuan	56926	1.1738	18	106	4	qinghai	4557	2.4948	49	15
5	guangxi	47691	1.4722	27	109	5	shanghai	7544	0.301	1	92
**Year 2019**											

1	guangdong	59194	1.0881	1	120	1	ningxia	2532	0.6904	16	11
2	hunan	55539	1.441	1	102	2	tianjin	3884	0.5022	9	16
3	sichuan	49144	1.1365	14	64	3	tibet	6267	4.4368	16	20
4	henan	42744	0.924	36	44	4	shanghai	6380	0.4359	14	69
5	xinjiang	40632	1.8407	22	556	5	beijing	6903	0.5892	20	45
Top rates						***Bottom rates***					
**Year 2005**											
1	tibet	2750	4.3972	50	21	1	shanghai	6090	0.6138	9	30
2	xinjiang	40020	2.9991	45	103	2	tianjin	3922	0.7082	36	17
3	hainan	8675	2.6941	9	34	3	beijing	8894	0.8038	32	73
4	guizhou	55891	2.6533	26	94	4	shandong	41935	0.8156	51	135
5	chongqing	41803	2.6449	14	104	5	liaoning	22810	1.0193	17	33
**Year 2009**											
1	tibet	3781	3.5575	1	21	1	shanghai	7492	0.403	1	86
2	xinjiang	40608	2.1208	17	141	2	tianjin	2965	0.4048	11	13
3	guizhou	61144	2.10945	18	124	3	beijing	8845	0.526	36	39
4	qinghai	5519	2.1078	19	22	4	shandong	44623	0.6903	1	91
5	hainan	8621	2.002	1	36	5	jiangsu	43901	0.8651	18	67
**Year 2014**											
1	tibet	2553	3.5854	16	14	1	shanghai	7544	0.301	1	92
2	qinghai	4557	2.4948	49	15	2	tianjin	3447	0.32	22	15
3	xinjiang	40777	2.194	11	121	3	beijing	8610	0.41025	22	43
4	guizhou	32026	1.9023	1	380	4	shandong	35141	0.5288	1	64
5	hainan	8094	1.6203	29	35	5	jiangsu	32863	0.5903	22	54
**Year 2019**											

1	tibet	6267	4.4368	16	20	1	shanghai	6380	0.4359	14	69
2	qinghai	7865	3.3941	40	25	2	tianjin	3884	0.5022	9	16
3	guizhou	36268	1.8519	1	82	3	shandong	27238	0.5044	1	48
4	xinjiang	40632	1.8407	22	556	4	jiangsu	24076	0.5547	49	34
5	hainan	7641	1.6738	14	33	5	beijing	6903	0.5892	20	45
